# The LCD-Composer webserver: high-specificity identification and functional analysis of low-complexity domains in proteins

**DOI:** 10.1093/bioinformatics/btac699

**Published:** 2022-10-25

**Authors:** Sean M Cascarina, Eric D Ross

**Affiliations:** Department of Biochemistry and Molecular Biology, Colorado State University, Fort Collins, CO 80523, USA; Department of Biochemistry and Molecular Biology, Colorado State University, Fort Collins, CO 80523, USA

## Abstract

**Summary:**

Low-complexity domains (LCDs) in proteins are regions enriched in a small subset of amino acids. LCDs exist in all domains of life, often have unusual biophysical behavior, and function in both normal and pathological processes. We recently developed an algorithm to identify LCDs based predominantly on amino acid composition thresholds. Here, we have integrated this algorithm with a webserver and augmented it with additional analysis options. Specifically, users can (i) search for LCDs in whole proteomes by setting minimum composition thresholds for individual or grouped amino acids, (ii) submit a known LCD sequence to search for similar LCDs, (iii) search for and plot LCDs within a single protein, (iv) statistically test for enrichment of LCDs within a user-provided protein set and (v) specifically identify proteins with multiple types of LCDs.

**Availability and implementation:**

The LCD-Composer server can be accessed at http://lcd-composer.bmb.colostate.edu. The corresponding command-line scripts can be accessed at https://github.com/RossLabCSU/LCD-Composer/tree/master/WebserverScripts.

## 1 Introduction

Low-complexity domains (LCDs) in proteins are regions in which a small subset of amino acids comprise an unusually large percentage of that region. LCDs that are enriched in different amino acids tend to be associated with distinct molecular functions and biophysical behavior, which may contribute to their functional specialization. Consequently, local enrichment of one or more specific amino acids provides a direct and intuitive way to identify/classify LCDs (Cascarina and Ross, 2018; Cascarina *et al.*, 2021b). Additionally, LCDs have been associated with a variety of human diseases including cancer (Lu *et al.*, 2021), neuromuscular disorders (Harrison and Shorter, 2017) and pathogenic infections (Cascarina and Ross, 2022; Davies *et al.*, 2017).

## 2 The LCD-Composer webserver

**Fig. 1.**
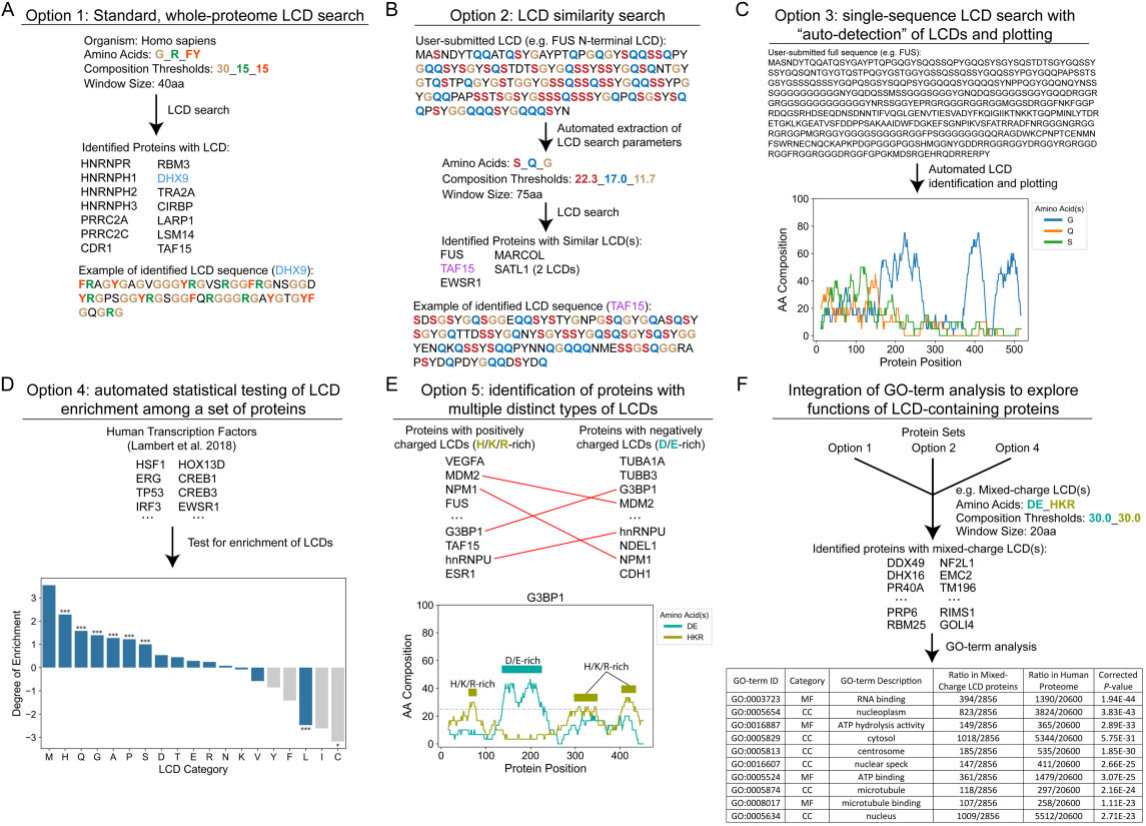
Examples of LCD-Composer analyses using each option

Our *l*ow-*c*omplexity *d*omain *compo*sition *s*cann*er* (LCD-Composer) offers user control over multiple search parameters, including sliding window size, amino acid(s) to use as defining features to identify LCDs, minimum composition thresholds associated with the defining amino acid(s) and minimum spacing of the defining amino acid(s). These parameters are described on the server ‘Help’ page and in Cascarina *et al.* (2021b). LCD searches can be based on simple criteria or on multiple criteria simultaneously (e.g. LCDs with combined serine/threonine composition ≥50% *and* combined phenylalanine/tryptophan/tyrosine composition ≥20%). LCD searches can be performed on UniProt reference proteomes or individual, user-defined proteins. The corresponding command-line scripts can be run on any FASTA-formatted proteome. The LCD-Composer server provides several options for performing LCD searches (Fig. 1), summarized in the sections below.

### 3.1 Option 1

Users can customize search parameters and perform an LCD search on a selected proteome, with the option of limiting the search to a single representative isoform for each protein or including all known isoforms for the corresponding organism (Fig. 1A). This option is equivalent to running the LCD-Composer command-line script.

### 3.2 Option 2

Users can submit a ‘query’ LCD sequence of their choosing: LCD-Composer then searches for compositionally similar LCDs by extracting search parameters from the query sequence (Fig. 1B). Users can choose the number of compositional features (with a maximum of four) to use as search parameters. Identified LCDs are automatically ranked according to compositional similarity to the user-submitted query sequence (defined as the normalized Manhattan distance between amino acid compositions of each identified LCD compared to the query LCD). LCD searches can be performed across organisms (e.g. a query LCD sequence from yeast can be used to search for similar LCDs in humans).

### 3.3 Option 3

Users can submit a single protein sequence and search for LCDs using customizable search parameters. This option is particularly useful when defining LCDs based on multiple simultaneous criteria, which can be challenging without a quantitative definition of domain boundaries. Additionally, this option offers automated, publication-quality plotting of amino acid composition as a function of protein position using our CompositionPlotter algorithm [(Cascarina *et al.*, 2021a); Fig. 1C].

### 3.4 Option 4

Users can submit a list of proteins and test for statistical enrichment/depletion of certain types of LCDs within that protein set. Enrichment tests can be specific to a user-defined LCD type (e.g. testing if Q/N-rich LCDs are enriched among transcription factors) or can be performed ‘naïvely’, where enrichment tests are automatically performed for the 20 canonical LCD classes [Fig. 1D; (Lambert *et al.*, 2018)].

### 3.5 Option 5

Proteins containing multiple distinct types of LCDs (‘co-occurring’ LCDs) can be specifically associated with certain functions (Cascarina *et al.*, 2021b). For example, users may be interested in proteins that contain both a positively charged LCD and a negatively charged LCD (Fig. 1E). After searching for multiple types of LCDs, users can submit the results of each search for comparison: only proteins that contain every type of user-defined LCD are returned to the user.

### 3.6 Automated GO-term analysis for LCD-containing proteins

Molecular functions of LCD-containing proteins can be remarkably LCD-type-specific (Cascarina *et al.*, 2021b), suggesting the existence of functional niches for each type of LCD. Therefore, the LCD-Composer webserver also offers automated gene ontology (GO)-term analysis that can be performed in conjunction with LCD searches (Fig. 1F), using the user-selected UniProt proteome to define the background set of proteins for enrichment analyses.

### 3.7 The LCD-Composer server complements existing LCD servers

Existing servers designed to identify LCDs or evaluate compositional biases in protein sequences include ProBias (Kuznetsov, 2008), LCR-eXXXplorer (Kirmitzoglou and Promponas, 2015), LCR-hound (Ntountoumi *et al.*, 2019), PlaToLoCo (Jarnot *et al.*, 2020), SAPS [(Brendel *et al.*, 1992); currently available at https://www.ebi.ac.uk/Tools/seqstats/saps] and Composition Profiler (Vacic *et al.*, 2007). In general, these servers excel at LCD identification and/or whole-protein composition analyses in the absence of pre-defined LCD features of interest. LCD-Composer complements these servers by enabling high-specificity LCD identification at a whole-proteome scale with pre-defined compositional features of interest or with search parameters calculated from a user-submitted query LCD sequence.

### 3.8 High-throughput analyses using command-line scripts

We also release command-line versions of each option available on the LCD-Composer server, which enable high-throughput analyses of multiple proteomes or LCD types, as well as analyses of alternative proteomes not supported by the server.
